# Up-regulation of L Antigen Family Member 3 Associates With Aggressive Progression of Breast Cancer

**DOI:** 10.3389/fonc.2020.553628

**Published:** 2021-01-21

**Authors:** Xubin Dong, Shihui Lv, Dianna Gu, Xiaohua Zhang, Zhiqiang Ye

**Affiliations:** ^1^Department of Thyroid and Breast Surgery, The First Affiliated Hospital of Wenzhou Medical University, Wenzhou, China; ^2^Department of Radiotherapy and Chemotherapy, The First Affiliated Hospital of Wenzhou Medical University, Wenzhou, China

**Keywords:** LAGE3, prognosis, lymph node metastasis, tumorigenesis, biomarker, triple-negative breast cancer

## Abstract

The role of L Antigen Family Member 3 (LAGE3) in breast cancer (BC) has not been sufficiently studied. In this study, we explored the clinical value and biological functions of LAGE3 in BC. Comprehensive analysis of LAGE3 was carried out on The Cancer Genome Atlas, Molecular Taxonomy of Breast Cancer International Consortium and Gene Expression Omnibus datasets. Results showed that LAGE3 expression was higher in BC tissues than in normal breast tissues of public datasets and our local cohort. Moreover, its expression was higher in BC patients with larger tumor size, significant lymph node metastasis, higher tumor grade, and more advanced disease stage. High expression of LAGE3 was correlated with poor prognosis, and LAGE3 could independently predict survival of BC patients. Functional enrichment analysis revealed a correlation between LAGE3 expression and biochemical metabolism and immune-related terms and cancer-related pathways. Analysis of tumor microenvironment indicated that LAGE3 expression was associated with the immune cell infiltration and anti-cancer immunity cycle. LAGE3 expression was higher in triple-negative breast cancer (TNBC) compared to hormone receptor-positive BC, but not HER2-positive subtype. Suppression of LAGE3 expression inhibited the proliferation and induced apoptosis of TNBC cell lines. Besides, the down-regulation of LAGE3 attenuated the migration and invasion but reduced the expression level of epithelial-mesenchymal-transition related proteins in TNBC cell lines. In conclusion, this study demonstrated for the first time that LAGE3 promotes the progression of BC. Therefore, it may be a potential diagnostic and prognostic biomarker, as well as a treatment target for BC.

## Introduction

Breast cancer (BC) is the most common malignant disease and the leading cause of cancer death among women worldwide. It has been estimated that about 276,480 new BC patients and 42,170 deaths occurred in the United States in 2020 (1). BC is staged based on the TNM (Tumor, Nodes, Metastasis) staging system, all of which characterize the disease, influence the prognosis, and guide the management of patients (2). The 5-year survival rates of BC patients with anatomical stages of stage I, IIA, IIB, IIIA, IIIB, and IV are 95, 85, 70, 52, 48, and 18%, respectively (3). Tumor size, lymph node metastasis (LNM), and histologic grade were identified as important prognostic factors in patients with BC (4–7). In early BC, the status of estrogen receptor (ER) and human epidermal growth factor receptor 2 (HER2) are the most well-established predictive markers for endocrine and HER2-directed therapies, respectively (8–10). As a most novel treatment modality for BC, immunotherapy is the complement of surgery, chemotherapy, radiotherapy, and targeted therapies (11, 12). Immune checkpoint inhibitors like Atezolizumab (anti-programmed death-ligand 1 antibody) have been approved for the treatment of BC (13). The tumor microenvironment (TME), consisting of various blood, immune, and stromal cells, is essential for tumor occurrence and development (14, 15). Despite tremendous research in these fields, there are still limited prognostic biomarkers that can be used in clinical practice. Thus, it is of great value to identify reliable BC-specific biomarkers for diagnosis, prognosis, and targeted therapy.

LAGE3, a gene belongs to the ESO/LAGE gene family, is ubiquitously expressed in many organs and cell types in humans. LAGE3 is also known as a component of the EKC/KEOPS complex, playing an important role in the regulation of RNA polymerase II-mediated positive transcription and tRNA threonyl carbamoyl adenosine metabolic process (16). Begik et al. identified LAGE3 as one of the top-ranked up-regulated RNA modification-related proteins in human cancers (17). In our previous research, we found that LAGE3 was a prognostic biomarker associated with levels of immune infiltration in colorectal cancer (CRC), clear cell renal cell cancer (ccRCC), and malignant pleural mesothelioma microenvironment (18–20). Goswami MT et al. had found that knockdown of LAGE3 significantly reduced cell proliferation in NSCLC cell lines (21). To our best knowledge, the clinical significance of LAGE3 and its distinct functions are yet to be fully elucidated in BC.

Bioinformatic analyses were performed in various public datasets and the local cohort to assess the expression of LAGE3 in BC tissues. Results showed that LAGE3 was a prognostic and diagnostic biomarker of BC. Additionally, we investigated the potential functional pathways associated with LAGE3 in BC. We then explored the association between LAGE3 and tumor microenvironment and different steps of the cancer-immunity cycle. Finally, we performed loss-of-function assays to explore the function of LAGE3 in BC cell lines. Our study suggested that LAGE3 might not only serve as a potential diagnostic and prognostic biomarker but also facilitate the progression of BC.

## Materials And Methods

### Bioinformatics

This study applied various publicly available BC datasets to perform a series of analyses that included exploration of the expression of LAGE3, inference of the composition of TME, and prediction of patient survival. For the TCGA dataset, RNA-seq, Mutation Annotation Format files, and clinical profiles of 1,093 human BC patients were identified, extracted, and validated from the Genomic Data Commons portal (22). This study utilized RNA-Seq by Expectation-Maximization (RSEM) expression values. For the Molecular Taxonomy of Breast Cancer International Consortium (METABRIC) database, 1,904 normalized microarray data and corresponding clinical profiles were obtained from the European Genome-Phenome Archive (accession number: EGAS00000000083) (23). We analyzed LAGE3 mRNA expression in BC and normal samples using the GSE5364 (24), GSE54002 (25), GSE42568 (26), GSE76250 (27), and GSE10810 (28) datasets as a validation in the GEO database. The Human Protein Atlas is a web-based tool that contains immunohistochemistry (IHC) based on pathological and detailed gene information on different types of tissues and cells (29, 30). In the present study, immunohistochemical staining was performed using the anti-LAGE3 antibody (Cat. No. HPA036122). We used STRING (https://string-db.org) to perform protein-protein interaction (PPI) network analysis.

The Kaplan–Meier plotter for breast cancer (31) is a web-based resource, contains multiple GEO datasets. We obtained the batch-effect removed LAGE3 expression profile and outcome of overall survival (OS), relapse-free survival (RFS), post-progression survival (PPS), disease-progression survival (PPS), and distant metastasis-free survival (DMFS) from Kaplan–Meier plotter (32). The Affymetrix ID 219061_s_at was used as the LAGE3 gene probe in this research. In the METABRIC dataset, OS and disease-specific survival (DSS) results were identified from the corresponding clinical profile. Uni- and multivariable Cox regressions model were built using the ”coxph” function in the “survival” package. Univariate analyses were only performed for the complete clinical parameters provided along with the respective LAGE3 expression data. Only significant factors with p-value <0.05 in univariate analyses were included in multivariable analyses.

The correlation analysis was assessed by function “cor.test” in R using the Spearman method. Genes which had highly ranked positive or negative correlation coefficients with LAGE3 were filtered. “enrichGo” and “enrichKEGG” functions in the “clusterprofiler” package were used to perform Gene Ontology (GO) and Kyoto Encyclopedia of Genes and Genomes (KEGG) analysis, respectively. “ggplot2” and “pheatmap” packages were used for visualization. The Gene set enrichment analysis (GSEA) was run using the “hallmark” signature collections from the Molecular Signature Database (MsigDB) (33).

The abundances of cell types from the RNA-seq matrix were estimated using tumor microenvironment analysis. For cell-type-specific analysis, the xCell algorithm was used to generate estimates for the relative proportions of various immune cell types. The xCell assigned enrichment scores across all samples for each cell type by integrating single-sample GSEA (ssGSEA) and deconvolution methods. Tracking the cancer immunity cycle is crucial for understanding the mechanisms of cancer immunity and provide significant clinical benefits for cancer immunotherapy (34, 35). The Tumor ImmunoPhenotype (TIP) pipeline was applied for immune activity profiling (36). This approach used a similar, ssGSEA-based approach to evaluate the relative activity of the seven steps of the immune cycle within bulk tumor samples.

### Patients and Breast Tissues Samples

Sixty pairs of breast tissues were obtained from the Department of Thyroid and Breast Surgery, The First Affiliated Hospital of Wenzhou Medical University. Further, all surgical specimens were independently confirmed by two experienced pathologists, and the results showed all sixty pairs of surgical specimens all met the diagnosis of BC and normal breast tissues, respectively. Collected fresh tissues were immediately snap-frozen in liquid nitrogen and stored at −80°C for further RNA was detected. The demographic and baseline characteristics of our local cohorts are shown in **Table 1**.

**Table 1:** Clinical and pathological features of patients in the WMU Breast cohort.

	Level	Overall
**N**		60
**Age (median [IQR])**		51.00 [44.00, 60.00]
**pT (%)**		
	T1	30 (50.0)
	T2	27 (45.0)
	T3	3 (5.0)
**pN (%)**		
	N0	32 (53.3)
	N1	16 (26.7)
	N2	10 (16.7)
	N3	2 (3.3)
**Stage (%)**		
	Stage1	20 (33.3)
	Stage2	29 (48.3)
	Stage3	11 (18.3)
**Lateral (%)**		
	Left	26 (43.3)
	Right	34 (56.7)
**Chemotherapy (%)**		
	No	11 (18.3)
	Yes	43 (71.7)
**Grade (%)**		
	G1	6 (10.0)
	G2	25 (41.7)
	G3	24 (40.0)
**Subtypes (%)**		
	HER2	12 (20.0)
	luminalA	9 (15.0)
	luminalB	21 (35.0)
	TNBC	18 (30.0)
**ER (%)**		
	Negative	29 (48.3)
	Positive	31 (51.7)
**PR (%)**		
	Negative	37 (61.7)
	Positive	23 (38.3)
**HER2 (%)**		
	Negative	45 (75.0)
	Positive	15 (25.0)
**ki67 (%)**		
	≤20%	20 (33.3)
	>20%	40 (66.7)

IQR, interquartile range.

### RNA Interference

siRNA for LAGE3 was obtained from RiboBio (Guangzhou, China) for cell interference. Based on the manufacturer’s protocol, Lipofectamine RNAiMAX transfection reagent (Thermo Fisher Scientific, USA) was mixed with siRNA to transfect cancer cell lines. Approximately 100,000 cells were seeded into one well of 6-well plates 24 h before transfection. The siRNA transfected concentrations of cancer cells were 75 nmol/ml per well. After 48–72 h, the transfected cells were harvested for subsequent qRT-PCR analysis. The negative control (si-NC) was not homologous to any human genomic sequence tracks. The sequences of the si-LAGE3 were as follows: si-LAGE3-1 sense: 5′-GTGGTTGGGAAGGATCTCA-3′, antisense: 5′-TGAGATCCTTCCCAACCAC-3′; si-LAGE3-2 sense: 5′-CGCTGGAAAGCTGAAGACT-3′, antisense: 5′-AGTCTTCAGCTTTCCAGCG-3′.

### Cell Cultures and Growth Conditions

All the cells used in the present study were all obtained from Shanghai Cell Biology, Institute of the Chinese Academy of Sciences (Shanghai, China). MDA-MB-231, Hs-578T, SK-BR-3, T-47D, and MCF-7 cells were cultured in DMEM (Gibco, USA) supplemented with 10% FBS (PAN Biotech, Germany). Roswell Park Memorial Institute 1640 medium (Gibco) supplemented with 10% FBS (Gibco) was used to culture BT-549 and BT-474 cells. MCF-10A cells were grown in MCF 10A cell-specific medium (CM-0525, Procell, China). All these cells were incubated in a humidified incubator at 37°C with 5% CO_2_.

### qRT-PCR

Total RNA was extracted from patient tissues and BC cell lines by TRIzol reagent (Invitrogen, USA). All RNA samples were temporarily stored at −80°C. The isolated RNA was measured at 260/280 nm to ensure the reliability of RNA quality and quantity. The 260/280 nm of our RNA ranged between 1.71 and 2.03. The ReverTra Ace qPCR RT Kit (Toyobo, Japan) was used for reverse transcription of the RNA. Real-time PCR was run and analyzed using the 7500 Fast quantitative PCR System (Applied Biosystems, USA). The relative expression of LAGE3 mRNA was presented using the method of 2−ΔΔCT with the endogenous control GAPDH to normalize the data. The nucleotide sequences of the primers used were followed: LAGE3 forward primer, 5′-GGATCTCACAGTGAGTGGCAGG-3′; LAGE3 reverse primer, 5′-GAAAGCTGGTCAAGAAAGTTGATG-3′; GAPDH forward primer, 5′-GTCTCCTCTGACTTCAACAGCG-3′; GAPDH reverse primer, 5′-ACCACCCTGTTGCTGTAGCCAA-3′.

### Cell Proliferation Assay

The Cell Counting Kit-8 (CCK-8, Beyotime, China) was used in this study to determine whether LAGE3 could affect the ability of cell proliferation. Forty-eight hours after siRNA transfection, 1,500 cells were seeded into each well of a 96-well plate with 100 ul medium supplemented with 10% FBS. At every indicated time point, the medium was replaced by 100ul of medium supplemented with CCK-8 (91 ul medium and 9 ul CCK-8). The cells were incubated at 37°C in 5% CO_2_ for 3 h. The optical density (OD) at a wavelength of 450 nm of each well was recorded on a microplate reader (SpectraMax Plus 384, Molecular Devices Corporation, CA, USA).

For colony formation assay, about 1,500 transfected cancer cells were added to each well in a 6-well plate and incubated for 10–14 days until colonies were formed. The plates were then gently washed by PBS and stained with crystal violet. Colony areas were determined by the Colony Area Plugin (37) in ImageJ software.

### Apoptosis Detection

Annexin-V-FITC apoptosis kit (BD Biosciences, Bedford, MD, USA) was used to determine the proportion of apoptotic cells. The cell apoptosis rates of MDA-MB-231 and BT-549 cells were analyzed after 48-h transfection with the LAGE3 siRNA and negative control. Transfected cells were collected from the 6-well plate and centrifuged three times at 800 rpm for 5 min each. After each centrifugation, cells were resuspended in 1 ml PBS. Cells were then resuspended in 500 μl binding buffer and stained with 5 μl Annexin V-FITC and PI. The results of the flow cytometry were analyzed by FlowJo (Tree Star, Ashland, OR, USA). The apoptosis rate was defined as the percentage of Q2 + Q3.

### Cellular Migration and Invasion Assay

Transwell plates (Corning, NY, USA) were used for the migration assay. Cells (4 × 104 cells/chamber) were seeded into the upper chamber, and the growth medium containing 10% FBS was added to the bottom chamber. After 24 h of incubation at 37°C, non-migrated cells on the upper chamber were carefully removed using a cotton swab. Cells migrating through the filter chamber were fixed with methanol and stained with 0.1% crystal violet. For the invasion assay, the same protocol was followed except that the chamber was Corning^®^ Matrigel^®^ invasion chamber (Corning Incorporated). Migrated or invaded cells were imaged in a 10× magnification microscope in five random fields for each well and quantified by ImageJ software.

### Western Blot

RIPA buffer (Solarbio, China) supplemented with protease inhibitors cocktail (Solarbio, China) and PMSF (Solarbio, China) were used to lyse the cells. The lysates were centrifuged at 12,600 × g. Total protein concentration was quantified using BCA analysis. The protein in the lysate was then separated using sodium dodecyl sulfate-polyacrylamide sodium gel electrophoresis (SDS-PAGE) and subsequently electrotransferred to a PVDF membrane (Millipore, Bedford, MA). The PVDF membrane was blocked with 5% skim milk (BD Biosciences, USA). The target proteins were detected by incubating the membrane at 4°C overnight with primary anti-LAGE3 antibody (#PA5-46520, Invitrogen) (1:1,000), anti-E-cadherin antibody (1:5,000, #ab40772, Abcam), anti-N-cadherin antibody (1:2,000, #66219-1-Ig, ProteinTech), anti-Vimentin antibody (1:2,000, #10366-1-Ig, ProteinTech) and primary anti-*β*-actin antibody (1:5,000, #66009-1-Ig, ProteinTech). Next, a secondary antibody goat anti-rabbit IgG (#ab97047, Abcam) was added to the membrane and incubated for 1 h at room temperature. Finally, the protein bands were visualized by the ECL detection kit (Beyotime Biotechnology, Shanghai, China). The bands were scanned and photographed by the UVP BioSpectrum AC image system (Upland, USA) and quantitated by ImageJ software.

### Statistical Analysis

Mann–Whitney U test or Student’s t-test were used in two group analyses. Comparisons between multiple groups were conducted by Kruskal–Wallis one-way analysis of variance (ANOVA). Survival rates were compared using the log-rank test, and hazard ratios were calculated by the Cox proportional hazards model. The Receiver operating characteristic (ROC) curve analysis was performed using the “pROC” package for the BC and adjacent normal breast tissues. The area under the ROC curve (AUC) value was estimated to investigate the diagnostic significance of LAGE3 expression in differentiating BC patients. R 3.6.1 and Graphpad Prism 8.1 were recruited according to analysis requirements.

## Results

### The Expression Level of LAGE3

**Figure 1:**
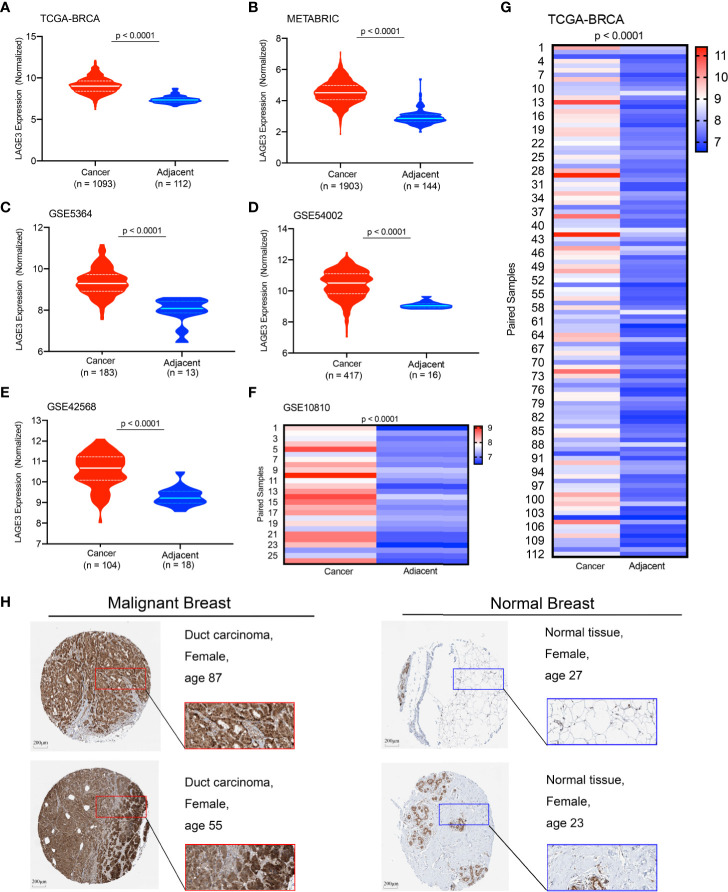
Expression profile of LAGE3 in malignant and normal breast tissues. The LAGE3 transcript levels in BC and adjacent normal breast tissues from the **(A)** TCGA-BRCA cohort; **(B)** METABRIC dataset; **(C)** GSE5364; **(D)** GSE54002; and **(E)** GSE42568 are presented as violin plots. **(F, G)** The heatmap was showing LAGE3 mRNA expression in matched BC and corresponding adjacent normal breast tissues in GSE10810 and TCGA cohorts. **(H)** Representative immunohistochemistry images and detailed information of LAGE3 in malignant and normal breast tissues were obtained from The Human Protein Atlas database.

The transcriptional profile of LAGE3 in BC and normal tissues was determined by analyzing different public datasets. Notably, LAGE3 mRNA level was significantly higher in BC than in normal breast tissues in the TCGA dataset, METABRIC, and multiple GEO datasets (all *p* < 0.0001, **Figures 1A–G**). We further examined the protein expression level of LAGE3 in BC and normal breast tissues based on the Human Protein Atlas database. LAGE3 protein levels in the BC tissues were higher than in normal breast tissues (**Figure 1H**). These results indicated that LAGE3 was over-expressed in BC.

### LAGE3 Could Serve as a Diagnostic Biomarker in BC Patients

**Figure 2:**
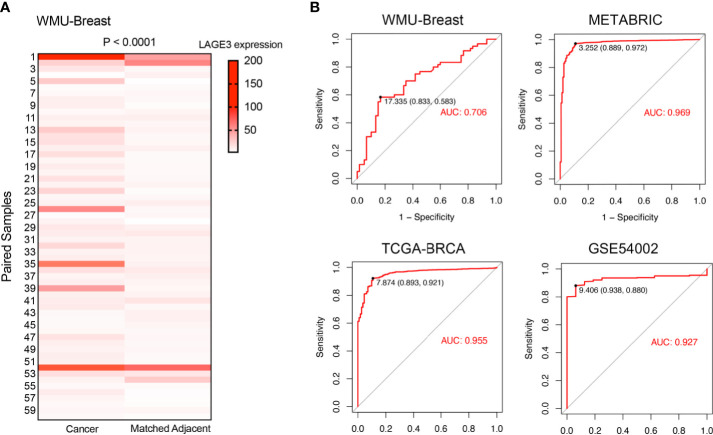
LAGE3 was a potential diagnostic biomarker in BC. **(A)** LAGE3 expression in local BC tissues and paired normal breast samples as determined by qRT–PCR. **(B)** ROC curve showing the diagnostic value of LAGE3 in BC patients from the local cohort, METABRIC, TCGA, and GSE54002 datasets.

In the local WMU Breast cohort, 60 BC and matched normal adjacent normal breast tissues were selected to validate the expression level of LAGE3 by qRT-PCR. As expected, LAGE3 mRNA levels were significantly greater in BC tissues than in normal breast tissues (*p* < 0.0001, **Figure 2A**). On the basis of these findings, we hypothesized that LAGE3 could be a diagnostic biomarker for BC. Analysis of ROC curve showed that LAGE3 exhibited diagnostic significance in the local cohort, with an AUC value of 0.706 (**Figure 2B**). Moreover, the METABRIC, TCGA, and GEO datasets were employed. The ROC curve analysis showed that the AUC value was 0.969, 0.955, and 0.927 in METABRIC, TCGA, and GSE54002 datasets, respectively. These results strongly indicated that LAGE3 could be a potential diagnostic biomarker for BC.

### LAGE3 Over-Expression Is Associated With a Range of Clinicopathological Factors in BC

**Figure 3:**
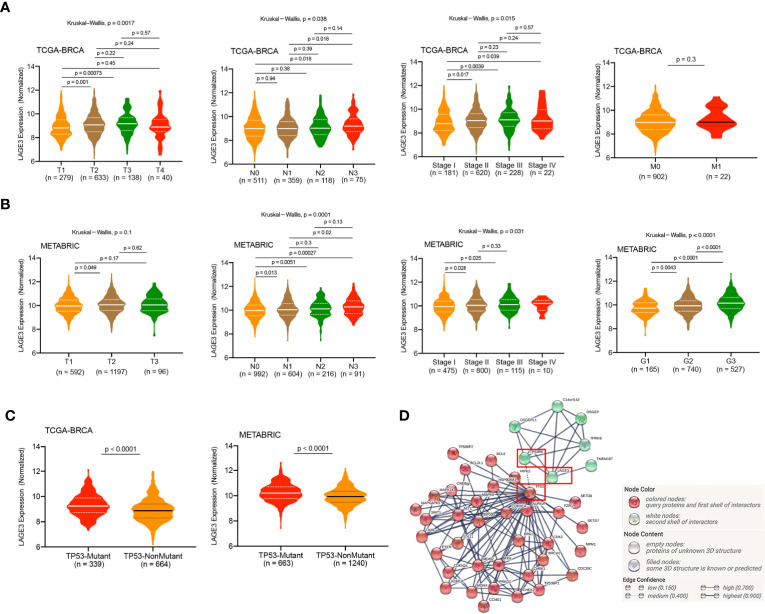
Correlation of LAGE3 expression with clinicopathological characteristics of BC patients. **(A)** LAGE3 expression in the different tumor sizes, lymph node metastasis, distant metastasis, and stage subgroups in the TCGA dataset. **(B)** LAGE3 expression in different tumor sizes, lymph node metastasis, tumor grade, and stage subgroups in METABRIC dataset. **(C)** LAGE3 expression level in wild-type and mutated TP53 groups. **(D)** PPI network of LAGE3, TP53, and their related genes. PPI, Protein**–**protein interaction.

We analyzed LAGE3 expression in BC specimens of different clinical and pathological factors in TCGA and METABRIC datasets. In the TCGA dataset, we observed an incremental change in the LAGE3 mRNA levels with larger tumor size, more metastatic lymph nodes, and higher disease stages (all *p* < 0.05, **Figure 3A**). As a validation, we found that there were statistically significant differences in LAGE3 expression between different LNM stages, tumor grades, and disease stages in the METABRIC dataset (all *p* < 0.05, **Figure 3B**). On analyzing the relationship between LAGE3 and the clinicopathological features in the local cohort, we found that large tumor size (*p* = 0.0127) was significantly associated with high LAGE3 expression. Furthermore, there was no significant difference in LAGE3 expression between different disease stages (**Supplementary Figure 1**).

TP53 mutant is in approximately 20**–**40% of all BC cases, and TP53 is the most frequently mutated gene in BC (38). Based on the important role of TP53 in the occurrence and development of BC, we compared the expression levels of LAGE3 between patients with and without TP53 mutants. In the TCGA and METABRIC datasets, patients with TP53 mutant had significantly higher LAGE3 expression than patients with TP53 wild type (all *p* < 0.0001, **Figure 3C**). Besides, an interaction network of LAGE3, TP53RK, and TP53 revealed potential crosstalk between LAGE3 and TP53-related proteins (**Figure 3D**).

### Prognostic Value of LAGE3

**Figure 4:**
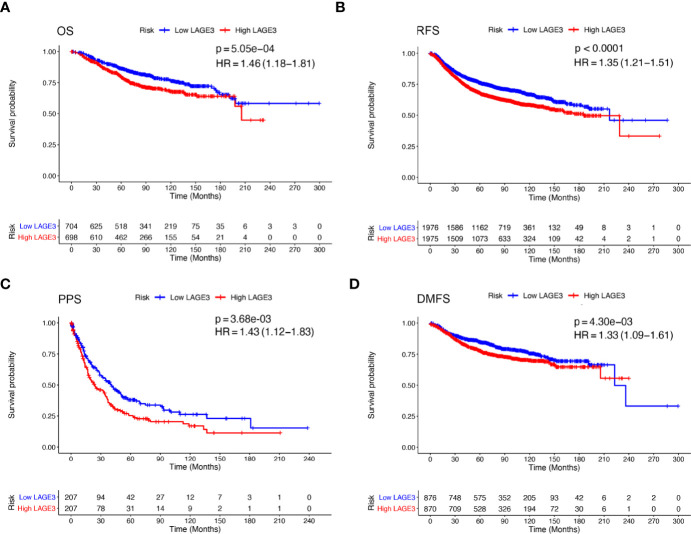
Prognostic value of LAGE3 in BC patients in K-M plotter database. **(A–D)** OS, RFS, PPS, and DMFS curve of high and low LAGE3-expressing BC patients. OS, overall survival; RFS, relapse-free survival; PPS, post-progression survival; DMFS, distant metastasis-free survival.

**Figure 5:**
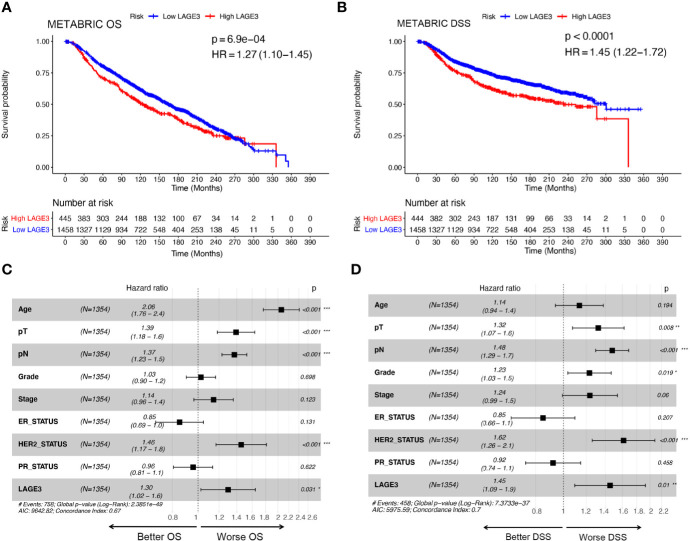
Prognostic value of LAGE3 in BC patients in the METABRIC cohort. **(A, B)** OS and RFS between LAGE3 high and low groups. Multivariate Cox regression analysis of LAGE3 expression for **(C)** OS and **(D)** DSS in BC patients. OS, overall survival; DSS, disease-specific survival.

To explore the prognostic value of LAGE3, correlation between LAGE3 mRNA level and disease outcome was assessed by the Kaplan**–**Meier plotter. As shown in **Figures 4A–D**, BC patients with high LAGE3 expression were associated with worse OS (log-rank *p* = 5.0e-04), RFS (log-rank *p* < 0.0001), PPS (log-rank *p* = 3.6e-03) and DMFS (log-rank *p* = 4.3e-03). We also examined the prognostic significance of LAGE3 in BC patients through the METABRIC dataset. We observed that both OS and DSS in patients with high LAGE3 group were shorter (**Figures 5A, B**, log-rank *p* = 6.9e-04 and *p* < 0.0001).

Subsequent univariate Cox regression analyses revealed that tumor size, LNM stage, tumor grade, disease stage, ER, PR, HER2, and LAGE3 expression were associated with OS and DSS (**Supplementary Tables 2**, **3**). On performing multivariate analysis of relevant clinical and pathological factors, we found that LAGE3 was an independent factor that could predict poor OS (*p* = 0.031, **Figure 5C**) and DSS (*p* = 0.01, **Figure 5D**) in BC. Tumor size, LNM stage, and status of HER2 were also identified as independent prognostic indicators for OS and DSS. These results supported previous findings, demonstrating that LAGE3 could independently predict survival in BC patients.

### Predicted Functions and Pathways

**Figure 6:**
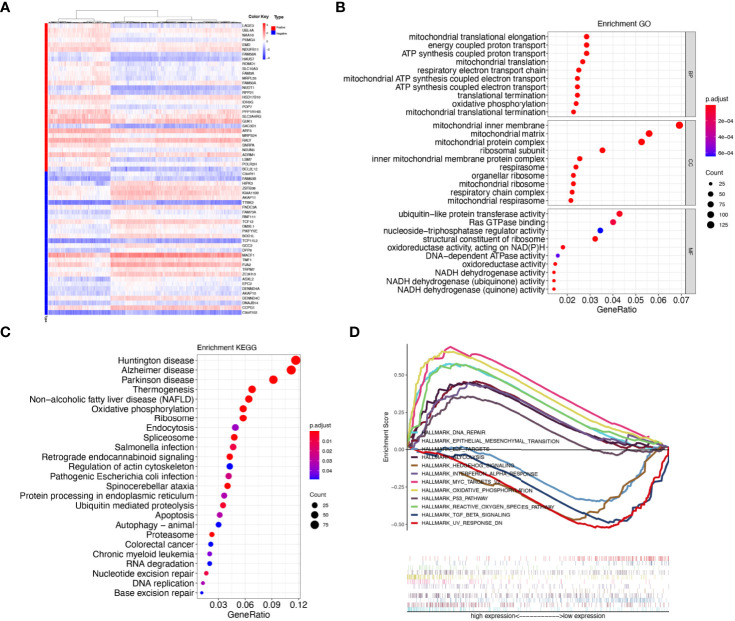
LAGE3 co-expressed genes and functional enrichment analysis. **(A)** Heatmaps showing the top 30 genes positively and negatively correlated with LAGE3. **(B)** GO enrichment analysis (top 10 terms of each subtype) and **(C)** KEGG enrichment analysis based on genes co-expressed with LAGE3. **(D)** GSEA analyses displayed key pathways associated with LAGE3 expression.

Genes which are co-regulated under specific biological state are more likely to be drivers of the underlying functions and pathways (39). For mining biological roles of LAGE3, we selected genes which had strong co-expression correlation (Spearman correlation value > 0.35 or < -0.35, *p* < 0.0001) with LAGE3 for functional enrichment analysis (**Figure 6A**). Additionally, GO and KEGG pathway enrichment analyses were performed on the co-expressed genes. The enrichment analysis of GO biological process indicated that these genes were involved in a variety of processes, including translational elongation, mitochondrial translation, and oxidative phosphorylation. GO cellular component revealed cellular structures, including mitochondrial inner membrane, mitochondrial matrix, and mitochondrial protein complex. GO molecular function included ubiquitin-protein transferase activity, Ras GTPase binding, and nucleoside-triphosphatase regulator activity (**Figure 6B**). Meanwhile, KEGG enrichment analysis demonstrated that LAGE3 was closely linked to neurodegenerative diseases (Huntington disease, Alzheimer’s disease, and Parkinson’s disease) and metabolism-related processes (Thermogenesis and oxidative phosphorylation) (**Figure 6C**).

For further comprehensive exploration of functions of LAGE3 in BC, we selected “hallmark gene sets” to perform gene set enrichment analysis (GSEA) based on LAGE3 expression. The GSEA results included: Cancer-related terms (such as epithelial-mesenchymal transition, hedgehog signaling, P53 pathway, and MYC targets V2; metabolism-related annotation (such as oxidative phosphorylation, reactive oxygen species pathway, and glycolysis); immune-related annotation (such as interferon-alpha response and TGF beta signaling) (**Figure 6D**). Taken together, these results suggested that LAGE3 might be linked with tumor development, metabolism, and immune activity in BC.

### LAGE3 Expression Correlates With Immune Infiltration and Cancer-Immunity Cycle

**Figure 7:**
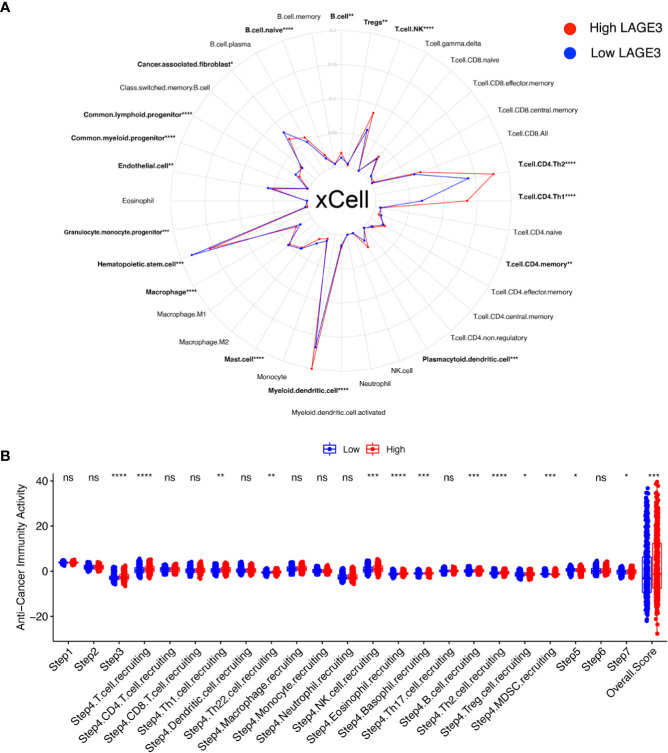
Correlation of LAGE3 with TME components and cancer-immunity cycle in BC. **(A)** Comparison of different TME components between high and low LAGE3 expression group. **(B)** Comparison of anti-cancer immunity scores of every cancer-immunity step between high and low LAGE3 expression group. ns, *p* ≥ 0.05; *p < 0.05; **p < 0.01; ***p < 0.001; ****p < 0.0001.

Immunotherapy is the newest and effective treatment for BC patients (40). Immune modulation of the TME is crucial for improving cancer immunotherapy (41). We evaluated the fraction of various types of cells in TME by xCell algorithm (**Figure 7A**). In particular, 16 differentially enriched cell types were significantly correlated with LAGE3 expression. For hematopoietic stem cell (HSC) group, common lymphoid progenitor, common myeloid progenitor, granulocyte monocyte progenitor, and hematopoietic stem cells were elevated in the low LAGE3 group. For lymphocytes, whole B cells, naive B cells, Natural killer T (NKT) cells, myeloid dendritic cells, Th1 cells, and Th2 cells showed a higher proportion while CD4+ memory T cells and Tregs showed a lower proportion in the high LAGE3 expression group (all *p* < 0.05). For myeloid non-lymphocytes, myeloid dendritic cells, plasmacytoid dendrite cells, and whole macrophages were increased, and mast cells were decreased in the high LAGE3 group. Stroma cells like endothelial cells and cancer-associated fibroblasts were elevated in the low LAGE3 group.

The cancer immune cycle initiates a series of gradual events and continues to expand. These steps enable the anti-cancer immune response to kill cancer cells effectively (42). We used TIP to visualize the activity of LAGE3 across the seven-step cancer-immunity cycle (**Figure 7B**). High LAGE3 group had higher anti-cancer immune scores in step 3 (*p* < 0.0001). We could observe that almost all high LAGE3 groups had higher anti-cancer immune scores in the trafficking of T cells to tumors (step 4). Recruiting of T cells, NK, eosinophil, basophil, Th1, Th2, Th22, and MDSC cells showed obvious differences among the two expression groups (all *p* < 0.05), while the process of infiltration of T cells into tumors (step 5) had lower anti-cancer immune scores in low LAGE3 group (*p* < 0.05). There was no significant difference between low and high LAGE3 groups in the release of cancer cell antigen (step 1), cancer antigen presentation (step 2), and recognition of cancer cells by T cells (step 6). In summary, the high LAGE3 group had a higher overall anti-cancer score than the low group (*p* < 0.001), and LAGE3 might play a positive role in the cancer-immunity cycle.

### Down-Regulation of LAGE3 Affects the Proliferation and Apoptosis Capacities of TNBC Cells

**Figure 8:**
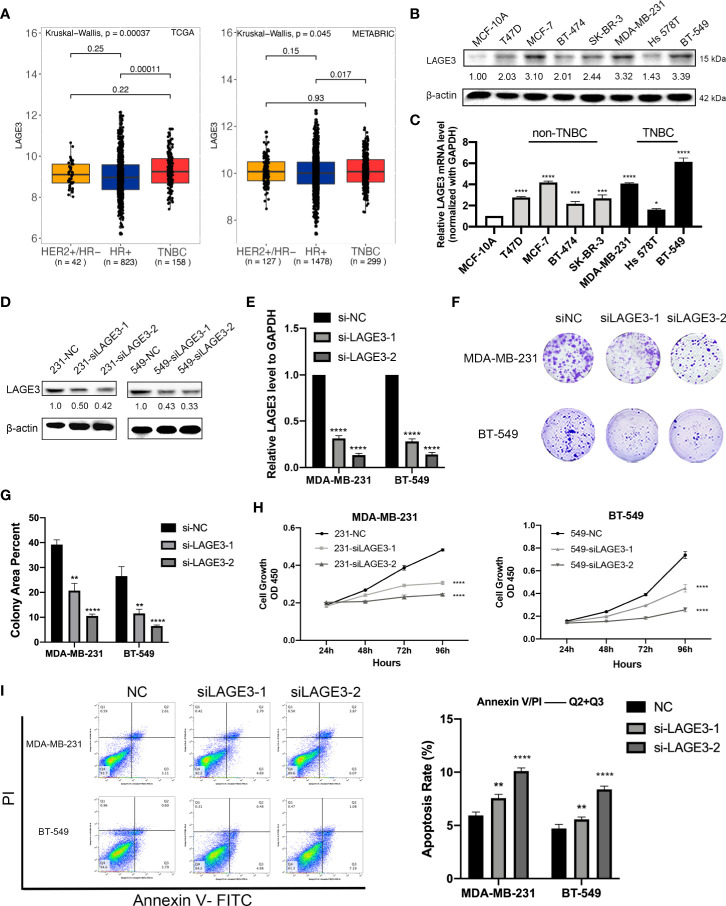
LAGE3 promoted proliferation and suppressed apoptosis of TNBC cell lines. **(A)** Box-and-whisker plot showing the expression of LAGE3 in various subtypes of BCs. **(B, C)** Protein and transcriptional level of LAGE3 in non-malignant MCF-10A cells and various BC cell lines. **(C, D)** Protein and mRNA level of LAGE3 in TNBC cell lines transfected with either negative control (si-NC) and LAGE3 targeting siRNA (si-LAGE3-1 or si-LAGE3-2). **(E, F)** Colony formation assay and **(G)** CCK-8 assay was showing the proliferation of cells transfected with si-NC or si-LAGE3. **(H)** Flow cytometry assay showing the number of apoptotic cells in different groups. Multiple groups were compared using unpaired Student’s t-test (two‐tailed). *p < 0.05; **p < 0.01; ***p < 0.001; ****p < 0.0001.

The results of the above analyses suggested that LAGE3 was over-expressed in BC and could independently predict prognosis, so we further explored its function in cell experiments. First, we explored the relationship between the LAGE3 expression and the intrinsic subtypes in BC. Our analysis revealed that expression of LAGE3 is significantly higher in triple-negative breast cancer (TNBC) when compared with hormone receptor-positive patients in TCGA (*p* = 0.0001) and METABRIC datasets (*p* = 0.017), but we didn’t find significant difference of LAGE3 expression between TNBC and HER2-positive subtype (**Figure 8A**). We also verified the up-regulation of LAGE3 in TNBC compared to corresponding normal tissue from (*p* = 0.0013, **Supplementary Figure 2A**) and our local cohort (*p* = 0.0139, **Supplementary Figure 2B**). Second, we found that LAGE3 expression was higher in BC cell lines than in non-tumorigenic cell line MCF-10A at both protein and transcription levels. TNBC cell lines, MDA-MB-231, and BT-549, which had relatively higher LAGE3 expression, were selected for further experiment (**Figures 8B, C**). The efficiency of LAGE3 down-regulation (transfected by si-NC and si-LAGE3) was examined in protein and transcription levels (**Figures 8D, E**). LAGE3 and TP53RK were both important components of the same complex, and the mutation state of TP53 was related to the expression of LAGE3. Thus, we boldly assumed that LAGE3 could regulate cellular processes, such as tumorigenesis and progression in BC. Cell proliferation and colony formation assays were then performed. The results revealed that down-regulated LAGE3 could effectively inhibit TNBC cell lines proliferation and colony formation by both si-LAGE3-1 and si-LAGE3-2 (**Figures 8F–H**).

From the KEGG analysis, we discovered that genes involved in apoptosis were significantly associated with LAGE3 expression. Therefore, we performed flow cytometry to detect apoptosis in TNBC cell lines after knocking down LAGE3. The apoptosis rate, calculated based on the total percentage of cells in the two right-side quadrants, was higher in the si-LAGE3 group than in the si-NC group in different TNBC cell lines (**Figure 8I**).

### LAGE3 Knockdown Suppresses Migration and Invasion in TNBC Cells

**Figure 9:**
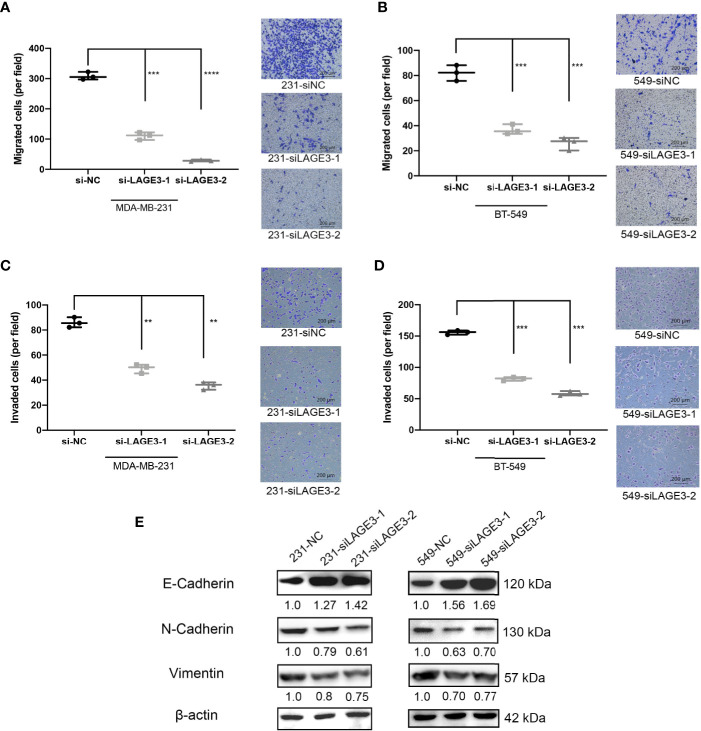
LAGE3 affected the migration and invasion of TNBC cell lines *via* EMT pathways. **(A–D)** The number of migrated or invaded cells in different groups as determined Transwell (scale bar = 200 μm). **(E)** The of EMT-related proteins in si-NC or si-LAGE3 cells as determined by Western blotting assay. All experiments were repeated at least three times. Group data were compared with unpaired Student’s t-test (two-tailed). EMT, epithelial–mesenchymal transition. **p < 0.01; ***p < 0.001; ****p < 0.0001.

Several studies have illustrated that migration and invasion of tumor cells contribute to cancer metastasis (43–45). Since we found LAGE3 expression to be correlated with LNM in BC patients, we performed Transwell migration assays and Matrigel invasion assays to assess the impact of LAGE3 on TNBC cell migration and invasion. The migration (**Figures 9A, B**) and invasion abilities of TNBC cell lines transfected with si-LAGE3 were significantly lower than those of cells transfected with si-NC (**Figures 9C, D**).

Several studies have revealed that epithelial-mesenchymal transition (EMT) is a crucial pathway, especially during TNBC proliferation and metastasis (46, 47). Mesenchymal markers, including Vimentin and N-cadherin, are indicators of the EMT and cancer metastasis. The switch from E-cadherin to N-cadherin is an important hallmark of EMT induction. The western blot assay revealed that the levels of Vimentin and N-cadherin decreased while the level of E-cadherin increased after the transfection of si-LAGE3-1 and si-LAGE3-2, implying that the EMT process was down-regulated (**Figure 9E**). Together, our findings suggest that LAGE3 might facilitate migration and invasion in TNBC cells.

## Discussion

BC is a heterogeneous group of diseases with different outcomes due to different molecular characteristics. Thousands of genes involved in controlling BC cell growth, death, and differentiation emphasize the importance of studying genes potentially associated with disease prognosis and progression. LAGE3 is a member of the tRNA-modifying EKC/KEOPS complex. However, the involvement of LAGE3 in BC has not been well characterized.

Previous studies reported that LAGE3 was significantly over-expressed in different types of cancer, compared to their corresponding normal tissues (17). We also found that up-regulated LAGE3 expression was associated with various malignant features in ccRCC (18, 19, 48). In the present study, we demonstrated that LAGE3 was highly up-regulated in BC tissues compared to normal breast tissues in six public datasets, which contained more than 3,500 patients. We then examined LAGE3 mRNA levels using our local validated cohort, including 60 matched BC and adjacent normal tissues. The results were consistent with those of the bioinformatic analysis. ROC curve analysis indicated that LAGE3 expression could robustly distinguish BC tissues from normal tissues. When we investigated the correlation between LAGE3 expression and clinicopathological factors in BC, we noticed that the up-regulation of LAGE3 was associated with the higher T stage, more metastatic lymph nodes, higher tumor grades, and more advanced disease stages. The expression of LAGE3 varied among patients with different TP53 mutation statuses, as it was elevated in BC patients with mutated TP53. Somatic mutations in TP53 were the most frequent events in BC and led to the inactivation of the gene and loss of tumor suppressor function (49). Since TP53 Regulating Kinase (TP53RK) and LAGE3 are the co-component of the EKC/KEOPS complex, we demonstrated the potential crosstalk between LAGE3, TP53RK, and TP53 by PPI network. All these results suggested LAGE3 is a potential biomarker for BC.

In our previous research, we found that LAGE3 up-regulation could independently predict poor prognosis in CRC and ccRCC patients (18, 19). For BC, the analysis of multiple BC cohorts indicated that high LAGE3 transcript expression was associated with poor prognosis. Moreover, we combined the existing clinical survival indicators and LAGE3 to perform univariate and multivariate Cox regression analysis in the METABRIC cohort. The result showed LAGE3 was an independent survival predictor for OS and DSS in BC. Accordingly, our data suggested that a high expression level of LAGE3 is associated with adverse patient outcomes, which are consistent with previous researches.

To promote the understanding of LAGE3 in the BC pathogenesis, we investigated possible mechanisms by which LAGE3 affects BC outcomes from a genomic perspective. Co-expressed genes usually act synergistically in biological processes under strict regulatory control, thus having an advantage in adaptive evolution (50). We identified some functional terms which corresponded to previous researches on LAGE3, especially the protein targeting and RNA modifications (17). LAGE3 was closely associated with several neurodegenerative diseases, which were also consistent with previous research (51). Besides, some cancer-related terms, such as EMT pathway, P53 pathway, and Hedgehog signaling pathway, were enriched in the result of KEGG and GSEA. Interestingly, metabolism-related terms like oxidative phosphorylation and immune-related terms like transforming growth factor-beta (TGF-*β*) signaling pathway were worth noticing. The TGF-*β* signaling pathway participates in various cancer processes such as cell proliferation, invasion, migration, angiogenesis, and apoptosis. It also controls metastasis-related factors, including EMT (52). Our results lead us to hypothesize that LAGE3 may participate in various biological processes in different diseases.

As the newest therapeutic method of BC, immunotherapy may become a key part of clinical cancer management (53). TME participates in BC progression before tumor cells invade into the stroma, and the importance of TME is increasingly being acknowledged (54). Our findings provide a detailed characterization of the association between LAGE3 and immune cells in TME. Daniel S. Chen et al. illustrated the seven steps of the cancer-immunity cycle, which is a process through which the immune system recognizes and kills cancer cells (42). The cancer-immunity cycle has become the basic framework for cancer immunotherapy research. Furthermore, we identified LAGE3 mRNA expression to be positively correlated with the anti-cancer immunity cycle. Further studies need to elucidate whether LAGE3 is a crucial factor in regulating TME and the cancer-immunity cycle.

Gene enrichment analysis results demonstrated that LAGE3 was significantly associated with EMT and apoptosis. We demonstrated that up-regulated LAGE3 expression was higher in TNBCs compared with the HR-positive subtype of BC. Functional validation of LAGE3 was performed in representative TNBC cell lines. First, we identified the potential role of LAGE3 in TNBC cell proliferation and apoptosis. Further, the migration and invasion-promoting effects of LAGE3 were investigated in TNBC cells. Compelling evidence shows that EMT enhances tumor initiation, progression, stemness, aggressiveness, and migration in various cancers (55–57). In BC, EMT occurs preferentially within a specific biological context, especially in basal-like or TNBC phenotypes (58). During the EMT process, epithelial marker E-cadherin is up-regulated, whereas mesenchymal markers, Vimentin, and N-cadherin, are down-regulated (59). Our study demonstrated that silencing LAGE3 up-regulates the expression of E-cadherin and down-regulates the expression of N-cadherin and Vimentin. These results indicated that LAGE3 promotes the progression of TNBC.

However, some limitations exist in this study. Firstly, the sample size used in our local cohort was small, and selection bias may exist. Secondly, the composition of TME and activity of the anti-cancer immunity cycle was inferred only based on transcriptional profiles. Follow-up studies with large sample size and using multidimensional omics data should examine specific biological processes and TME heterogeneity in BC tissues. Lastly, the mechanisms through which LAGE3-regulated tumorigenesis and metastasis in TNBC were not clarified. In the future, *in vivo* models are advocated to explore detailed biological mechanisms of LAGE3 in TNBC.

In summary, this study demonstrates that the LAGE3 is over-expressed in BC and can be a reliable diagnostic and prognostic predictor in patients with BC. In addition, we reveal that LAGE3 regulates TME and the cancer-immunity cycle. Lastly, the results indicate that LAGE3 promotes TNBC cell growth, migration, and invasion and suppress apoptosis. LAGE3 may regulate the progression of TNBC *via* the EMT pathway. We hope these findings will boost the discovery of biomarkers, which will enhance the accuracy of treatment and improve the prognosis of BC patients.

## Data Availability Statement

The original contributions presented in the study are included in the article/**Supplementary Material**. Further inquiries can be directed to the corresponding authors.

## Ethics Statement

All research protocols have been approved and implemented through the ethical standards of the institutional review board of the First Affiliated Hospital of Wenzhou Medical University (Approval No. 2012-57). The patients/participants provided their written informed consent to participate in this study.

## Author Contributions

XD contributed to study design, bioinformatic analysis, molecular biology experiments, and manuscript draft. SL contributed to patient sample preparation. DG, XZ, and ZY contributed to the revision of the manuscript. All authors contributed to the article and approved the submitted version.

## Funding

This study was supported by the funding of the National Natural Science Foundation of China (No. 81802328), Wenzhou Science and Technology Plan Project (No. Y20180846 and No. Y20180214), and Young Talents Program of the First Affiliated Hospital of Wenzhou Medical University (No. qnyc094).

## Conflict of Interest

The authors declare that the research was conducted in the absence of any commercial or financial relationships that could be construed as a potential conflict of interest.

## Supplementary Material

Supplementary Table 1 Clinical and pathological features of patients in TCGA and METABRIC cohort.

Supplementary Figure 1 Correlation of LAGE3 expression with clinicopathological characteristics in local BC patients. **(A, B)** LAGE3 expression in the **(A)** different tumor sizes and **(B)** stage subgroups in the TCGA dataset.

Supplementary Figure 2 Expression profile of LAGE3 in TNBC tissues and normal breast tissues. **(A)** The LAGE3 transcript levels in TNBC and adjacent normal breast tissues from the GSE76250 cohort. **(B)** LAGE3 expression in local TNBC tissues and paired normal breast samples as determined by qRT–PCR.

## References

[1] Siegel RL Miller KD Jemal A Cancer statistics, 2020. CA: A Cancer J Clin (2020) 70(1):7–30. 10.3322/caac.21590 31912902

[2] Gress DM Edge SB Greene FL Washington MK Asare EA Brierley JD Principles of cancer staging. AJCC cancer staging manual (2017) 8:3–30.

[3] Newman LA ed. Epidemiology of locally advanced breast cancer. Semin Radiat Oncol (2009) 19:195–203. 10.1016/j.semradonc.2009.05.003 19732683

[4] Koscielny S Tubiana M Le M Valleron A Mouriesse H Contesso G Breast cancer: relationship between the size of the primary tumour and the probability of metastatic dissemination. Br J Cancer (1984) 49(6):709–15. 10.1038/bjc.1984.112 PMC1976833 6733019

[5] Carter CL Allen C Henson DE Relation of tumor size, lymph node status, and survival in 24,740 breast cancer cases. Cancer (1989) 63(1):181–7. 10.1002/1097-0142(19890101)63:1<181::AID-CNCR2820630129>3.0.CO;2-H 2910416

[6] Rakha EA El-Sayed ME Lee AH Elston CW Grainge MJ Hodi Z Prognostic significance of Nottingham histologic grade in invasive breast carcinoma. J Clin Oncol (2008) 26(19):3153–8. 10.1200/JCO.2007.15.5986 18490649

[7] de Boer M van Dijck JA Bult P Borm GF Tjan-Heijnen VC Breast cancer prognosis and occult lymph node metastases, isolated tumor cells, and micrometastases. J Natl Cancer Institute (2010) 102(6):410–25. 10.1093/jnci/djq008 20190185

[8] Perou CM Sørlie T Eisen MB Van De Rijn M Jeffrey SS Rees CA Molecular portraits of human breast tumours. nature (2000) 406(6797):747–52. 10.1038/35021093 10963602

[9] Sørlie T Perou CM Tibshirani R Aas T Geisler S Johnsen H Gene expression patterns of breast carcinomas distinguish tumor subclasses with clinical implications. Proc Natl Acad Sci (2001) 98(19):10869–74. 10.1073/pnas.191367098 PMC58566 11553815

[10] Yu K Lee CH Tan PH Tan P Conservation of breast cancer molecular subtypes and transcriptional patterns of tumor progression across distinct ethnic populations. Clin Cancer Res (2004) 10(16):5508–17. 10.1158/1078-0432.CCR-04-0085 15328190

[11] Esteva FJ Hubbard-Lucey VM Tang J Pusztai L Immunotherapy and targeted therapy combinations in metastatic breast cancer. Lancet Oncol (2019) 20(3):e175–e86. 10.1016/S1470-2045(19)30026-9 30842061

[12] Narayan P Wahby S Gao JJ Amiri-Kordestani L Ibrahim A Bloomquist E FDA Approval Summary: Atezolizumab plus paclitaxel protein-bound for the treatment of patients with advanced or metastatic TNBC whose tumors express PD-L1. Clin Cancer Res (2020) 26:2284–9. 10.1158/1078-0432.CCR-19-3545 32001481

[13] Schmid P Adams S Rugo HS Schneeweiss A Barrios CH Iwata H Atezolizumab and nab-paclitaxel in advanced triple-negative breast cancer. New Engl J Med (2018) 379(22):2108–21. 10.1056/NEJMoa1809615 30345906

[14] Place AE Huh SJ Polyak K The microenvironment in breast cancer progression: biology and implications for treatment. Breast Cancer Res (2011) 13(6):1–11. 10.1186/bcr2912 PMC3326543 22078026

[15] Soysal SD Tzankov A Muenst SE Role of the tumor microenvironment in breast cancer. Pathobiology (2015) 82(3-4):142–52. 10.1159/000430499 26330355

[16] Faranda S Frattini A Zucchi I Patrosso C Milanesi L Montagna C Characterization and fine localization of two new genes in Xq28 using the genomic sequence/EST database screening approach. Genomics (1996) 34(3):323–7. 10.1006/geno.1996.0293 8786131

[17] Begik O Lucas MC Liu H Ramirez JM Mattick JS Novoa EM Integrative analyses of the RNA modification machinery reveal tissue-and cancer-specific signatures. Genome Biol (2020) 21:1–24. 10.1186/s13059-020-02009-z PMC7204298 32375858

[18] Dong X Lv S Zhang X Hao R Upregulation of LAGE3 correlates with prognosis and immune infiltrates in colorectal cancer: A bioinformatic analysis. Int Immunopharmacol (2020) 85:106599. 10.1016/j.intimp.2020.106599 32438075

[19] Dong X Gu D-N Wang O Ye Z LAGE3 correlates with tumorigenic immune infiltrates in the clear cell renal cell carcinoma microenvironment. Int Immunopharmacol (2020) 87:106793. 10.1016/j.intimp.2020.106793 32683301

[20] Dong X Song J He M Sun J Tao E Zhang X Identification of the prognostic and immunotherapeutic potential of L antigen family member 3 in malignant pleural mesothelioma. Clin Trans Med (2020) 10(7):e207. 10.1002/ctm2.207 PMC7689948 33252853

[21] Goswami MT VanDenBerg KR Han S Wang LL Singh B Weiss T Identification of TP53RK Binding Protein (TPRKB) dependency in TP53-deficient cancers. Mol Cancer Res (2019) 17:1652–64. 10.1158/1541-7786.MCR-19-0144 PMC6679750 31110156

[22] Network CGA Comprehensive molecular portraits of human breast tumours. Nature (2012) 490(7418):61. 10.1038/nature11412 23000897 PMC3465532

[23] Curtis C Shah SP Chin S-F Turashvili G Rueda OM Dunning MJ The genomic and transcriptomic architecture of 2,000 breast tumours reveals novel subgroups. Nature (2012) 486(7403):346–52. 10.1038/nature10983 PMC3440846 22522925

[24] Yu K Ganesan K Tan LK Laban M Wu J Zhao XD A precisely regulated gene expression cassette potently modulates metastasis and survival in multiple solid cancers. PLoS Genet (2008) 4(7):e1000129. 10.1371/journal.pgen.1000129 18636107 PMC2444049

[25] Tan TZ Miow QH Miki Y Noda T Mori S Huang RYJ Epithelial-mesenchymal transition spectrum quantification and its efficacy in deciphering survival and drug responses of cancer patients. EMBO Mol Med (2014) 6(10):1279–93. 10.15252/emmm.201404208 PMC4287932 25214461

[26] Clarke C Madden SF Doolan P Aherne ST Joyce H O’driscoll L Correlating transcriptional networks to breast cancer survival: a large-scale coexpression analysis. Carcinogenesis (2013) 34(10):2300–8. 10.1093/carcin/bgt208 23740839

[27] Liu Y-R Jiang Y-Z Xu X-E Hu X Yu K-D Shao Z-M Comprehensive transcriptome profiling reveals multigene signatures in triple-negative breast cancer. Clin Cancer Res (2016) 22(7):1653–62. 10.1158/1078-0432.CCR-15-1555 26813360

[28] Pedraza V Gomez-Capilla JA Escaramis G Gomez C Torné P Rivera JM Gene expression signatures in breast cancer distinguish phenotype characteristics, histologic subtypes, and tumor invasiveness. Cancer: Interdiscip Int J Am Cancer Soc (2010) 116(2):486–96. 10.1002/cncr.24805 20029976

[29] Uhlen M Zhang C Lee S Sjöstedt E Fagerberg L Bidkhori G A pathology atlas of the human cancer transcriptome. Science (2017) 357(6352):eaan2507. 10.1126/science.aan2507 28818916

[30] Uhlén M Fagerberg L Hallström BM Lindskog C Oksvold P Mardinoglu A Tissue-based map of the human proteome. Science (2015) 347(6220):1260419. 10.1126/science.1260419 25613900

[31] Györffy B Lanczky A Eklund AC Denkert C Budczies J Li Q An online survival analysis tool to rapidly assess the effect of 22,277 genes on breast cancer prognosis using microarray data of 1,809 patients. Breast Cancer Res Treat (2010) 123(3):725–31. 10.1007/s10549-009-0674-9 20020197

[32] Nagy Á Lánczky A Menyhárt O Győrffy B Validation of miRNA prognostic power in hepatocellular carcinoma using expression data of independent datasets. Sci Rep (2018) 8(1):9227. 10.1038/s41598-018-29514-3 29907753 PMC6003936

[33] Liberzon A Birger C Thorvaldsdóttir H Ghandi M Mesirov JP Tamayo P The molecular signatures database hallmark gene set collection. Cell Syst (2015) 1(6):417–25. 10.1016/j.cels.2015.12.004 PMC4707969 26771021

[34] Vonderheide RH Domchek SM Clark AS Immunotherapy for breast cancer: what are we missing? Clin Cancer Res (2017) 23:2640–6. 10.1158/1078-0432.Ccr-16-2569 PMC5480967 28572258

[35] Pusztai L Karn T Safonov A Abu-Khalaf MM Bianchini G New strategies in breast cancer: immunotherapy. Clin Cancer Res (2016) 22(9):2105–10. 10.1158/1078-0432.CCR-15-1315 PMC9359478 26867935

[36] Xu L Deng C Pang B Zhang X Liu W Liao G TIP: a web server for resolving tumor immunophenotype profiling. Cancer Res (2018) 78(23):6575–80. 10.1158/0008-5472.CAN-18-0689 30154154

[37] Guzman C Bagga M Kaur A Westermarck J Abankwa D ColonyArea: an ImageJ plugin to automatically quantify colony formation in clonogenic assays. PLoS One (2014) 9(3):e92444. 10.1371/journal.pone.0092444 24647355 PMC3960247

[38] Børresen-Dale AL TP53 and breast cancer. Hum Mutat (2003) 21(3):292–300. 10.1002/humu.10174 12619115

[39] Jia Z Liu Y Guan N Bo X Luo Z Barnes MR Cogena, a novel tool for co-expressed gene-set enrichment analysis, applied to drug repositioning and drug mode of action discovery. BMC Genomics (2016) 17(1):414. 10.1186/s12864-016-2737-8 27234029 PMC4884357

[40] Emens LA Breast cancer immunotherapy: facts and hopes. Clin Cancer Res (2018) 24(3):511–20. 10.1158/1078-0432.CCR-16-3001 PMC5796849 28801472

[41] Devaud C John LB Westwood JA Darcy PK Kershaw MH Immune modulation of the tumor microenvironment for enhancing cancer immunotherapy. Oncoimmunology (2013) 2(8):e25961. 10.4161/onci.25961 24083084 PMC3782527

[42] Chen DS Mellman I Oncology meets immunology: the cancer-immunity cycle. Immunity (2013) 39(1):1–10. 10.1016/j.immuni.2013.07.012 23890059

[43] van Zijl F Krupitza G Mikulits W Initial steps of metastasis: cell invasion and endothelial transmigration. Mutat Res/Rev Mutat Res (2011) 728(1-2):23–34. 10.1016/j.mrrev.2011.05.002 PMC4028085 21605699

[44] Kramer N Walzl A Unger C Rosner M Krupitza G Hengstschläger M In vitro cell migration and invasion assays. Mutat Res/Rev Mutat Res (2013) 752(1):10–24. 10.1016/j.mrrev.2012.08.001 22940039

[45] Justus CR Leffler N Ruiz-Echevarria M Yang LV In vitro cell migration and invasion assays. JoVE (J Visualized Experiments) (2014) 88:e51046. 10.3791/51046 PMC4186330 24962652

[46] Heerboth S Housman G Leary M Longacre M Byler S Lapinska K EMT and tumor metastasis. Clin Trans Med (2015) 4(1):6. 10.1186/s40169-015-0048-3 PMC4385028 25852822

[47] Salt MB Bandyopadhyay S McCormick F Epithelial-to-mesenchymal transition rewires the molecular path to PI3K-dependent proliferation. Cancer Discovery (2014) 4(2):186–99. 10.1158/2159-8290.CD-13-0520 24302555

[48] Dong X Yang Q Gu J Lv S Song D Chen D Identification and validation of L Antigen Family Member 3 as an immune-related biomarker associated with the progression of papillary thyroid cancer. Int Immunopharmacol (2020) 90:107267. 33310661 10.1016/j.intimp.2020.107267

[49] Martin A Kanetsky P Amirimani B Colligon T Athanasiadis G Shih H Germline TP53 mutations in breast cancer families with multiple primary cancers: is TP53 a modifier of BRCA1? J Med Genet (2003) 40(4):e34–e. 10.1136/jmg.40.4.e34 PMC1735423 12676907

[50] Niehrs C Pollet N Synexpression groups in eukaryotes. Nature (1999) 402(6761):483–7. 10.1038/990025 10591207

[51] Braun DA Rao J Mollet G Schapiro D Daugeron M-C Tan W Mutations in KEOPS-complex genes cause nephrotic syndrome with primary microcephaly. Nat Genet (2017) 49(10):1529 10.1038/ng.3933 28805828 PMC5819591

[52] Gupta SC Kim JH Prasad S Aggarwal BB Regulation of survival, proliferation, invasion, angiogenesis, and metastasis of tumor cells through modulation of inflammatory pathways by nutraceuticals. Cancer Metastasis Rev (2010) 29(3):405–34. 10.1007/s10555-010-9235-2 PMC2996866 20737283

[53] Pestalozzi BC Zahrieh D Mallon E Gusterson BA Price KN Gelber RD Distinct clinical and prognostic features of infiltrating lobular carcinoma of the breast: combined results of 15 International Breast Cancer Study Group clinical trials. J Clin Oncol (2008) 26(18):3006–14. 10.1200/JCO.2007.14.9336 18458044

[54] Ma X-J Dahiya S Richardson E Erlander M Sgroi DC Gene expression profiling of the tumor microenvironment during breast cancer progression. Breast Cancer Res (2009) 11(1):R7. 10.1186/bcr2222 19187537 PMC2687710

[55] Jayachandran A Dhungel B Steel JC Epithelial-to-mesenchymal plasticity of cancer stem cells: therapeutic targets in hepatocellular carcinoma. J Hematol Oncol (2016) 9(1):74. 10.1186/s13045-016-0307-9 27578206 PMC5006452

[56] Tsai JH Yang J Epithelial–mesenchymal plasticity in carcinoma metastasis. Genes Dev (2013) 27(20):2192–206. 10.1101/gad.225334.113 PMC3814640 24142872

[57] Singh A Settleman J EMT, cancer stem cells and drug resistance: an emerging axis of evil in the war on cancer. Oncogene (2010) 29(34):4741–51. 10.1038/onc.2010.215 PMC3176718 20531305

[58] Sarrió D Rodriguez-Pinilla SM Hardisson D Cano A Moreno-Bueno G Palacios J Epithelial-mesenchymal transition in breast cancer relates to the basal-like phenotype. Cancer Res (2008) 68(4):989–97. 10.1158/0008-5472.CAN-07-2017 18281472

[59] Ackland ML Newgreen DF Fridman M Waltham MC Arvanitis A Minichiello J Epidermal growth factor-induced epithelio-mesenchymal transition in human breast carcinoma cells. Lab Invest (2003) 83(3):435–48. 10.1097/01.LAB.0000059927.97515.FD 12649344

