# Supplementary figures and images for: Up-regulation of L Antigen Family Member 3 Associates With Aggressive Progression of Breast Cancer
Source: Front Oncol. 2021 Jan 21;10:553628. doi: 10.3389/fonc.2020.553628 (PMC7858652; doi:10.3389/fonc.2020.553628)

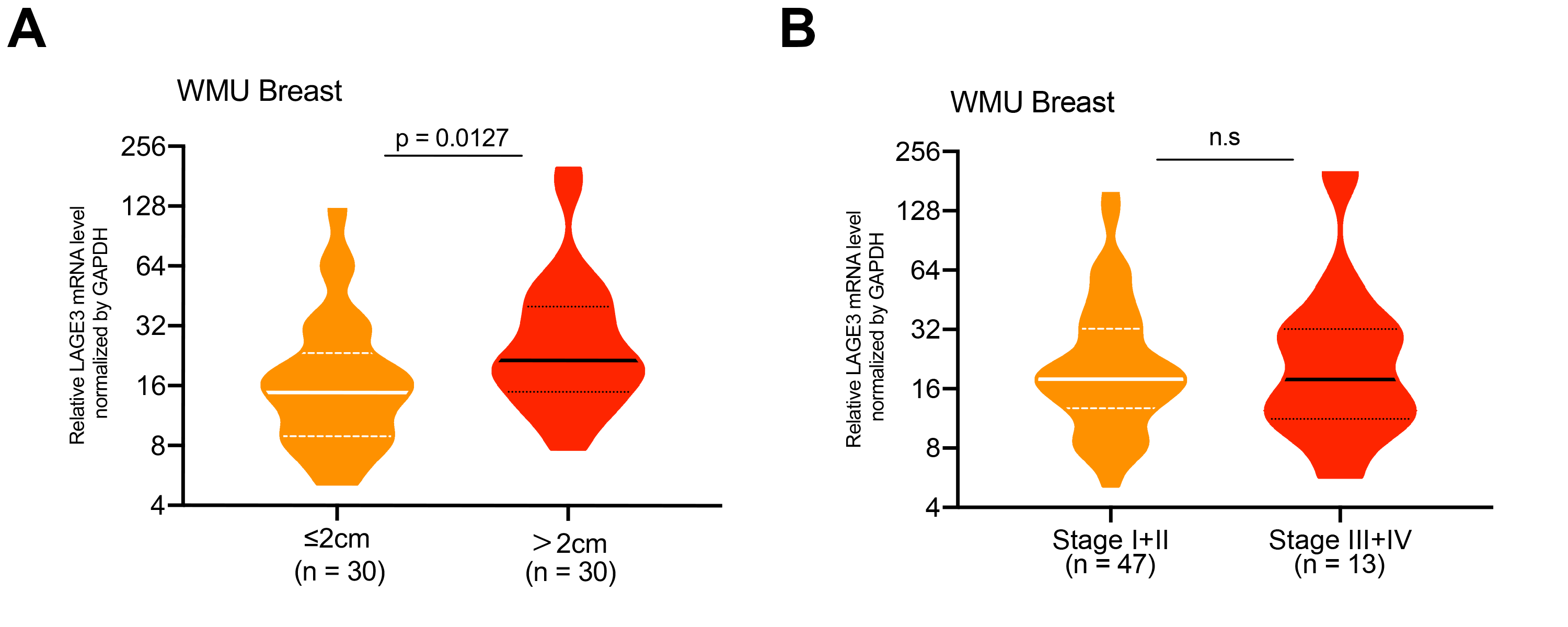

Supplement: Supplementary Figure 1 — Correlation of LAGE3 expression with clinicopathological characteristics in local BC patients. (A, B) LAGE3 expression in the (A) different tumor sizes and (B) stage subgroups in the TCGA dataset. [file Image_1.tiff]

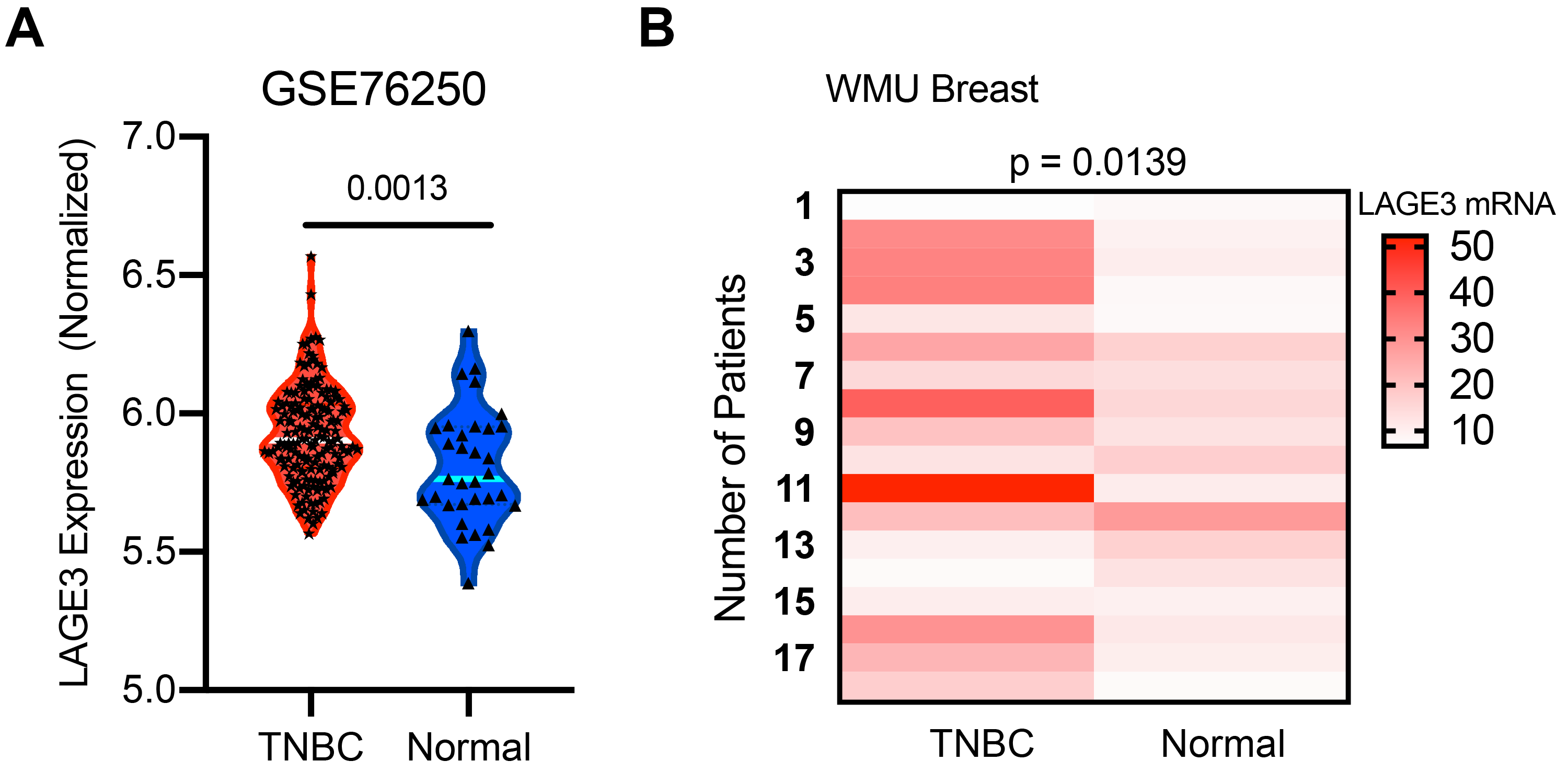

Supplement: Supplementary Figure 2 — Expression profile of LAGE3 in TNBC tissues and normal breast tissues. (A) The LAGE3 transcript levels in TNBC and adjacent normal breast tissues from the GSE76250 cohort. (B) LAGE3 expression in local TNBC tissues and paired normal breast samples as determined by qRT–PCR. [file Image_2.tiff]
